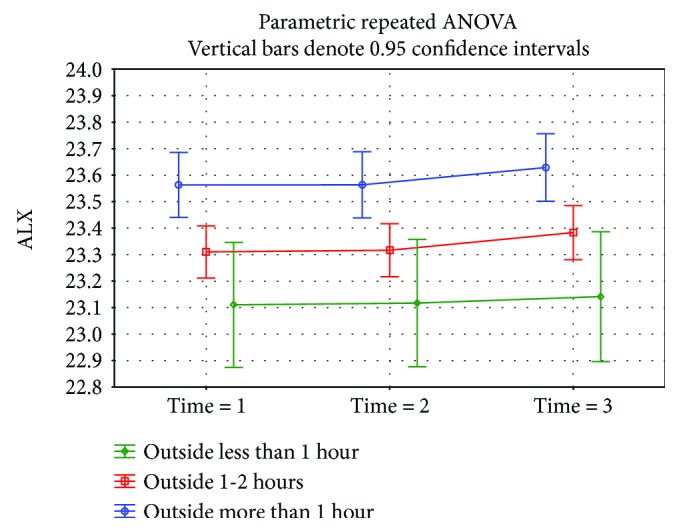# Myopia Progression Risk: Seasonal and Lifestyle Variations in Axial Length Growth in Czech Children

**DOI:** 10.1155/2018/5076454

**Published:** 2018-03-06

**Authors:** Stepan Rusnak, Vaclav Salcman, Lenka Hecova, Zdenek Kasl

**Affiliations:** ^1^Department of Ophthalmology, University Hospital in Pilsen, Alej Svobody 80, 304 60 Pilsen, Czech Republic; ^2^Faculty of Education, University of West Bohemia, Veleslavínova 42, 306 14 Pilsen, Czech Republic

## Abstract

The growth in the prevalence of myopia leads to the growth of socioeconomic stress in society. It is important to detect any potential risk factors leading to myopia onset and progression. Among the potential risk factors, the lack of natural daylight exposure and the lack of the physical activity together with excess of near-work activities in children are the most prevalent. In the study, the axial length growth depending on the season and the type of behaviour was measured. The assessment was performed in 12-year-old children, 398 eyes of whom were included and measured during the winter and summer period. The children were categorized by the amount of time spent on near-work, physical, and outdoor activity. *Results*. Statistically significantly higher (*p* < 0.0001) axial length growth was observed during the winter period. Statistically significantly (*p* < 0.0001) more frequently, the eyeball growth has been proved during the winter season. According to the way of spending leisure time, no statistically significant difference was reported within the individual subgroups in the development of the eyeball length during the observed period. However, statistically significant differences were ascertained in the eyeball initial length within various groups. *Conclusion*. The lack of daylight exposure may lead to myopia progression.

## 1. Introduction

The prevalence of myopia is growing worldwide, and myopia is becoming a major epidemiological problem. In 2000, according to the latest studies, 1406 million people (i.e., 22.9% of the world's population) suffered from myopia, and 163 million people (i.e., 2.7% of the world's population) suffered from high myopia. In 2050, a total of 4758 million people worldwide (49.8% of the world's population) are expected to be myopic, and 938 million people (9.8% of the world's population) are expected to suffer from high myopia [1]. The prevalence of this refractive error varies according to age, ethnicity, and geographical locality [2].

High myopia is associated with comorbidities that increase the risk of severe and irreversible loss of vision, such as dense cataract, retinal detachment, subretinal neovascularization, and glaucoma [3–5]. Growth in the prevalence of myopia leads to the growth of socioeconomic stress in society.

It is very important to look for the reasons for the growth of myopia prevalence in the population and to find ways to reduce the occurrence of myopia and its progression due to the increase of myopia occurrence in society, as well as the potential severe health and social consequences. According to the outcomes of the studies carried out in recent years, lifestyle can influence the onset and progression of myopia. Physical activity and stays in daylight have a possible protective effect [6–8], while near work is a risk factor [9, 10].

As previously mentioned, the prevalence of myopia is dependent upon a number of factors. Therefore, we decided to map this situation in the Central European population of children in the age range of 11 to 17 years. Biometric examinations of both eyes and examinations of central visual acuity of both eyes are performed at regular, six-month intervals. At the same time, questionnaire surveys are carried out, oriented towards the method of spending leisure time. We concentrate primarily on the development of the axial length of the eyeball at particular periods of time and on the presence of any risk, or protective factors affecting the growth of the axial length of the eyeball. Our research is conducted in cooperation with specialists involved in physical education and prevention, who evaluate the ascertained data on a continual basis and prepare preventive programs for preschool-aged children.

## 2. Materials and Methods

### 2.1. Methodology

A prospective single-centre study was initiated in the spring of 2016 (April‐May). The children enrolled in this study are examined at six-month intervals. For the purposes of this paper, we used the data from 3 initial examinations (time 1—spring 2016, time 2—autumn 2016, and time 3—spring 2017), and according to the plan, the study will not be finished earlier than in the spring of 2019.

The examination starts with the determination of the best-uncorrected central visual acuity of both eyes. The visual acuity is measured with ETDRS logMAR charts, at a distance of 4 metres; the room lighting intensity is between 50–100 foot-candles of light. During the examination, the schoolchild is sitting and is without correction (that means without spectacles or contact lenses), and the other eye is covered with an eye patch or pad.

Subsequently, the ocular biometry is measured using the optical biometry technique. In our case, the ocular biometry consists of determining the axial length (ALX, measured in millimetres, the final value is an average of 3 measurements) and anterior chamber depth (ACD, measured in millimetres, the final value is an average of 5 measurements) of both eyes. The measurements are provided in a noncontact way with IOL Master equipment from Zeiss.

Afterwards, the questionnaire survey is done.

The eye history of children is checked on a regular basis—primarily the previous history of eye surgeries, wearing of spectacles for vision correction, and its possible changes. In addition, the questionnaire focuses on the method of spending leisure time after classes and necessary home study and homework. The goal is to determine how many hours a day children spend outdoors during daylight, the amount of time spent on sports, and the amount of time spent on near work (reading, work on a mobile phone or tablet) and on the computer.

### 2.2. Statistical Methods

The statistical analysis was carried out using the SAS Institute Inc., Cary, NC, USA, and SW Statistics programs (StatSoft Inc., Tulsa, OK, USA).

The basic statistical data, such as mean values, standard deviation, variance, median, minimum, and maximum values, were calculated for the measured parameters in the whole research sample, as well as in individual groups and subgroups. Frequencies were tested for categorical variables. Selected statistical figures were also graphically processed to so-called box and whisker plot diagrams, histograms, and pie charts.

To compare the differences in the distribution of individual parameters between various groups, we used a nonparametric analysis of variance (Wilcoxon two-tailed test). The development of the eye length over time was tested using the Wilcoxon paired test and Friedman ANOVA analysis. The differences in categorical variables between the studied groups were tested using the chi-square test and Fisher exact test. With the aid of odds ratio expression, specificity, and sensitivity, we searched for an optimal cut-off value of various factors in relation to the eye length growth. The repeated ANOVA test was used to evaluate the development of the eye length in relation to various factors, such as the amount of time spent reading, on the computer, outdoors, or on sports. The relationships between the studied parameters were described using the Spearman correlation coefficient and expressed using the linear regression (least-square method).

The statistical significance was determined at the boundary of alpha = 5% (0.05).

### 2.3. Patient Group

The study involves 264 children, junior students of grammar schools in the city of Pilsen—a homogenous group from one geographical location (Pilsen), Caucasian race, and exposed to a similar level of environmental pollutants, with similar diet habits. On account of lack of data (absence of children at the time of any examination, failure to complete examination due to photophobia, etc.), 66 children were removed from our statistics.

At the time of initiation of the study, our research sample consisted of 396 eyes of 198 children in the age range of 11 to 13 years (median of 12 years). The research sample consisted of 43.4% boys and 56.6% girls.

The research sample was divided into various subgroups, according to the method of spending leisure time. 9.6% of children spend time outdoors less than 1 hour, 55% spend 1 to 2 hours, and 35.4% spend more than 2 hours. 7.1% of children are not involved in any sporting activities, 47% spend 3 hours a day or less on sporting activities, and 46% do sports more than 3 hours a day. 53% of the research sample spent less than 3 hours a day with near work and 47% spent 3 hours and more. 60.6% of children spend less than 3 hours a day on the computer and 39.4% spend 3 hours and more.

## 3. Results and Discussion

### 3.1. Results

**Figure 1:**
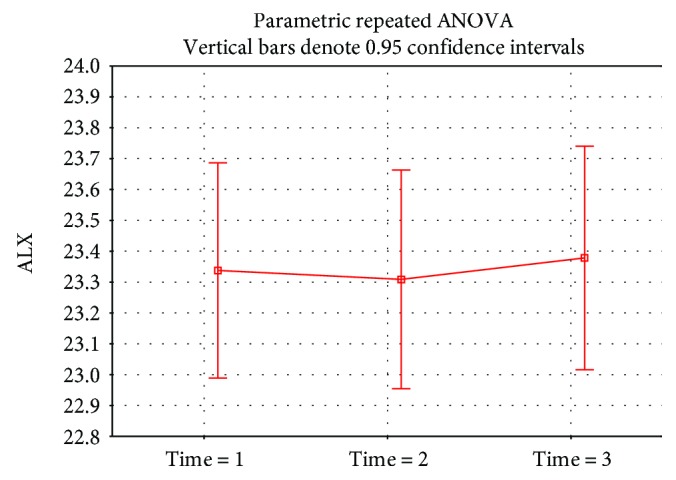


In the statistical evaluation of the project pilot part, we focused on the development of the eye length in two periods between time 1 and time 2 (i.e., between spring and autumn 2016—summer season) and time 2 and time 3 (i.e., between autumn 2016 and spring 2017—winter season). The median length of the eyeball was 23.34 mm at time 1 (range of 21.15 to 23.73 mm, 75th percentile, 23.85 mm), 23.37 mm at time 2 (range of 21.15 to 26.02 mm, 75th percentile, 23.83), and 23.43 mm at time 3 (range of 21.11 to 26.25 mm, 75th percentile, 23.91 mm). Statistically significantly higher (*p* < 0.0001) axial length growth during the winter period was observed (Figure 1).

**Figure 2:**
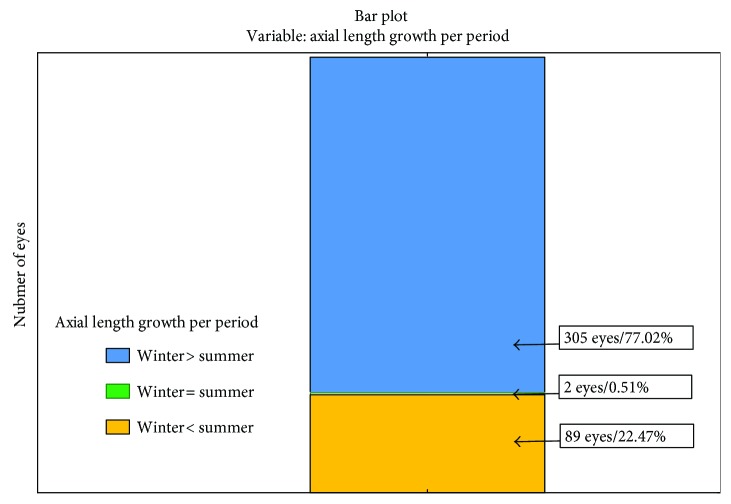


Statistically significantly (*p* < 0.0001) more frequently, the eyeball growth during the winter season has been proved. In 77.02% of eyes, the axial length growth during the winter season was observed, contrary to 22.47% during the summer season (Figure 2).

According to the method of spending leisure time, no statistically significant difference was reported within the individual subgroups in the development of eyeball length during the observed period, but statistically significant differences were ascertained (using the parametric repeated ANOVA analysis) in the eyeball initial length (time 1) within various groups.

**Figure 3:**
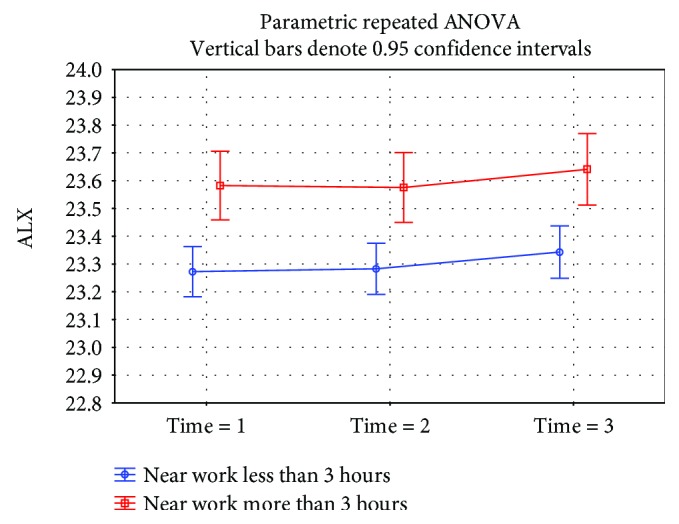


**Figure 4:**
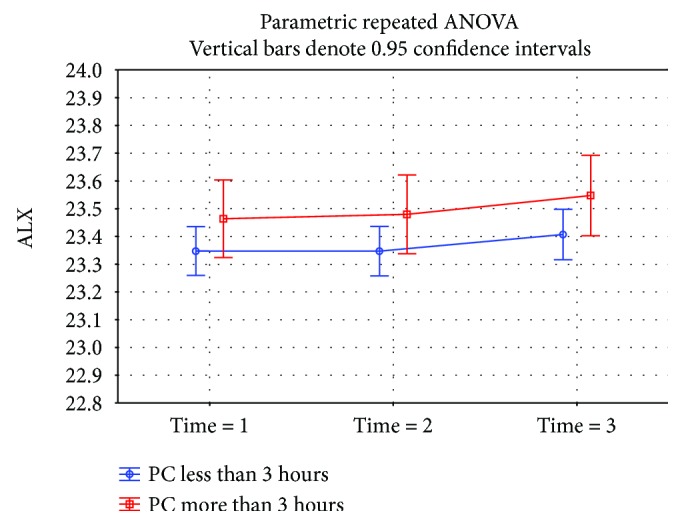


**Figure 5:**
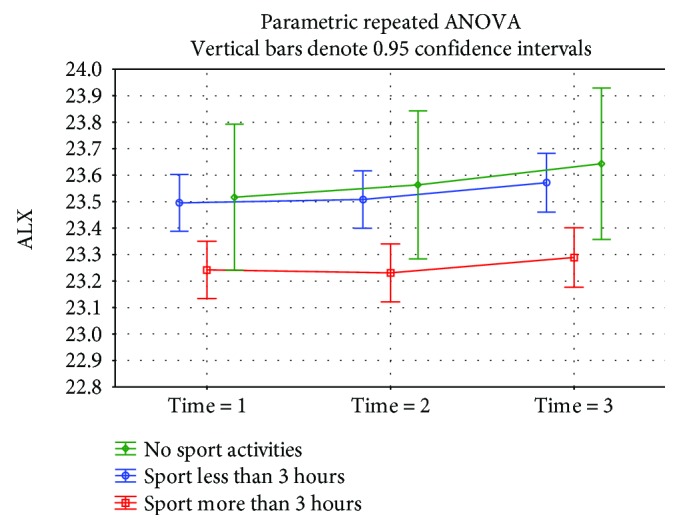


A statistically significant difference (*p* = 0.0002) was demonstrated in the eyeball length at time 1 in children doing near-work activities for almost 3 hours a day or more (23.57 mm median) than children who were involved in this type of work for less than 3 hours a day (23.29 mm median) (Figure 3). A not statistically significantly (*p* = 0.18) higher eyeball length at time 1 was found in the group working on the computer for 3 hours per day and more (23.49 mm median) versus the group involved in this type of activity less than 3 hours (23.31 mm median) (Figure 4). The eyeball length at time 1 was statistically significantly (*p* = 0.002) lower in the group of children involved in sporting activities for 3 hours and more (23.21 mm median), children involved in sporting activities less than 3 hours, respectively, children not involved in any sporting activities where the median of the initial eyeball length was 23.56 mm, respectively, 23.51 mm (Figure 5). The highest eyeball length at time 1 (23.53 mm median) was in the group of eyes of probands spending their time outdoors for 2 hours and more; the lowest eyeball length was in the group of probands spending their time outdoors for 1 hour a day or less (23.29 mm median); and in the group spending their time outdoors for 1-2 hours a day, the median eyeball length was 23.34 mm (Figure 6). The differences in between these groups were statistically significant (*p* = 0.002).

Age and gender did not have a statistically significant effect on the eyeball length at any of the tested times.

## 4. Discussion

Myopia is known to be influenced by both genetic and environmental factors. The educational level, near-work overload, daylight exposure, and physical activity are among the most cited [11]. The results of our research demonstrate that the eyeballs of our probands grew more during the winter season. The seasonal effect on the progression of the eyeball length in myopia in Chinese children is confirmed, for example, by Donovan et al. [12], and the similar conclusions were found by Fulk et al. [7] in North American school children. Similarly, as the authors of the said papers, we assume that the main reason of this state is a generally lower exposure to natural daylight and less amount of time spent outdoors in the winter season.

In the Czech Republic, summer holidays last 2 months (July and August), while winter holidays last only 2 weeks (1 week in December and 1 week between February and mid-April). The Czech Republic is between 48 and 51 degrees of north latitude. At 50 degrees of north latitude, the daylight lasts 13 hours and 30 minutes up to 16 hours and 15 minutes in the period of July and August, and, for example, the daylight lasts only 8 hours and 5 minutes up to 8 hours and 15 minutes during the Christmas holiday season in December [13]. That means that during several weeks of winter season, school children are not exposed to daylight on working days.

Our paper also points out that a different growth of the eyeball length, in dependence of the method of spending leisure time, occurs in children before the start of study at grammar schools, that is, before 11 to 13 years of age. Within the framework of preventive programs, it will be necessary to focus on preschool-age children and juniors at primary schools.

Sporting activities, as a preventive factor against excessive eyeball growth, have been proven to be effective if these activities are performed for 3 hours a day, or more. By contrast, near work and computer work could be a risk factor for increased eyeball growth if children spend 3 hours a day or more with these activities (outside the framework of school teaching and school duties). Contrary to our assumptions and the results of other work [6], our study did not show any protective effect of staying outdoors. As a possible cause of this discrepancy, we consider the fact that the length of stay outdoors in general was ascertained regardless of the carried-out activities; therefore, the total length of outdoor stay also counts the time the children spend on near work (e.g., reading, working on a tablet/mobile phone, etc.).

Our study also showed that no statistically significant difference occurred within 1 year in the growth of the eyeball length in comparison to individual groups with different risk, or protective factors. We can assume therefore that the influence of these factors is long lasting and cumulates over time. In the next phase of our study, we will therefore focus on determining the minimum time of influence of these factors that is necessary to capture the statistically significant changes in the growth of the axial eyeball length. Katuzny and Koszewska-Kolodziejczak [14] point out to the fact that the proportional eye growth is developed in myopic eyes until the age of 14, and the growth in the axial length is significantly accelerated after this age. The study will continue at least until spring 2019, and we consider it extending to spring 2023 (i.e., until the time when the probands will achieve the age of 18 to 20) in order to capture the period when stopping or accelerating the eyeball growth occurs.

## 5. Conclusions

Children and adult myopia is a considerable worldwide problem with the prevalence of myopia increasing over recent decades, and expressive growth could also be expected in the future. Discovering the causes of the development and progression of myopia in the children population and designing preventive programs should belong among the priorities of ophthalmologists and paediatricians.

The preschool age, or the start of school attendance, should be critical for the initiation of preventative programs. Already, at the time of entering grammar schools, the differences in eyeball length are statistically significant, depending on the daily regimens of the children. Our study demonstrates the impact of regular sporting activities as a preventative factor against the eyeball growth; therefore, active and regular sporting activities should be included in preschool and school education, and children should be motivated to regularly perform these activities also after classes. Our paper also points to a negative influence of near work and a possible negative influence of work on the computer for 3 hours a day, or more. The use of state-of-the-art technology and means such as tablets, computers, and so on is a frequent teaching trend already as early as in preschool education. Due to the significant burden of children with these devices after school too, regular use of these technologies should be carefully considered in classrooms, and children and their parents should be thoroughly instructed about the negative impacts of their overuse in near work and computer work on the development of eyeball length and the possible progression of myopia.

To determine the critical age where the negative or protective impact of the individual factors under consideration begins to manifest, further work needs to be carried out.

## Figures and Tables

**Figure 6:**